# Excited-State Lifetime of NV Centers for All-Optical Magnetic Field Sensing

**DOI:** 10.3390/s24072093

**Published:** 2024-03-25

**Authors:** Ludwig Horsthemke, Jens Pogorzelski, Dennis Stiegekötter, Frederik Hoffmann, Lutz Langguth, Robert Staacke, Christian Laube, Wolfgang Knolle, Markus Gregor, Peter Glösekötter

**Affiliations:** 1 Department of Electrical Engineering and Computer Science, FH Münster—University of Applied Sciences, Stegerwaldstr. 39, 48565 Steinfurt, Germany; pogorzelski.jens@fh-muenster.de (J.P.); dennis.stiegekoetter@fh-muenster.de (D.S.); frederik.hoffmann@fh-muenster.de (F.H.); peter.gloesekoetter@fh-muenster.de (P.G.); 2 Quantum Technologies GmbH, Alte Messe 6, 04103 Leipzig, Germany; 3 Leibniz Institute of Surface Engineering (IOM), Permoserstr. 15, 04318 Leipzig, Germany; 4 Department of Engineering Physics, FH Münster—University of Applied Sciences, Stegerwaldstr. 39, 48565 Steinfurt, Germany; markus.gregor@fh-muenster.de

**Keywords:** nitrogen-vacancy center, quantum sensor, fluorescence lifetime, all-optical, magnetometry

## Abstract

We investigate the magnetic field-dependent fluorescence lifetime of microdiamond powder containing a high density of nitrogen-vacancy centers. This constitutes a non-intensity quantity for robust, all-optical magnetic field sensing. We propose a fiber-based setup in which the excitation intensity is modulated in a frequency range up to 100MHz. The change in magnitude and phase of the fluorescence relative to B=0 is recorded where the phase shows a maximum in magnetic contrast of $5.8^{\circ }$ at 13MHz. A lock-in amplifier-based setup utilizing the change in phase at this frequency shows a 100 times higher immunity to fluctuations in the optical path compared to the intensity-based approach. A noise floor of $20\;\mu T/\sqrt{\mathrm{Hz}}$ and a shot-noise-limited sensitivity of $0.95\;\mu T/\sqrt{\mathrm{Hz}}$ were determined.

## 1. Introduction

The use of negatively charged nitrogen-vacancy (NV) centers in diamonds has attracted considerable attention in the recent past, especially in the field of magnetic field sensing. Approaches using microwave (MW) excitation achieve high sensitivities [1,2] and spatial resolutions [3,4] while operating at room temperature. They are, however, limited in their application due to the necessity of MW delivery, which typically requires a galvanic connection. Additionally, the MW delivery adds complexity and may have adverse effects, like local heating or the creation of eddy currents. In contrast, all-optical approaches simplify the sensor design as a step toward industrial application. They rely on the fluorescence change caused by spin mixing for magnetic fields up to ~50mT achieving sensitivities of 14–50 $\mu T/\sqrt{\mathrm{Hz}}$ [5,6,7,8,9]. Other all-optical setups utilize the ground-state level-anticrossing, reporting noise floors of $0.45\;\mathrm{nT}/\sqrt{\mathrm{Hz}}$, but requiring high precision in angular alignment and a bias magnetic field [10,11,12]. Furthermore, the cross-relaxation features near zero magnetic field in high-NV-density samples have been investigated in MW-free setups [13,14], reporting estimated photon shot-noise-limited sensitivities of $4.5\;\mathrm{nT}/\sqrt{\mathrm{Hz}}$ [14]. They can be implemented using fiber optics [7,13,15,16] to construct a non-magnetic, high-insulation resistance probe and potentially be used in harsh environments. These designs are, however, still subject to challenges, like movement in the optical fiber or laser intensity fluctuations. In setups that only observe the fluorescence intensity, these would be misinterpreted as magnetic field changes. One possible solution is the use of a non-intensity quantity like the fluorescence lifetime, which shows a relative change, similar to the fluorescence intensity, upon application of a magnetic field [3,17].

In this work, a sample composed of an ensemble of microdiamonds is first characterized in a time-correlated single-photon counting (TCSPC) setup with regard to the magnetic field-dependent fluorescence lifetime. Afterward, a setup is presented in which the material is fixed to the tip of an optical fiber, which is used for excitation and fluorescence collection. The magnitude and phase of the fluorescence upon excitation with a varying frequency in the range up to 100MHz is recorded, and its dependence on magnetic fields is analyzed. Furthermore, the phase at one specific excitation frequency is used for magnetic field sensing and analyzed concerning its immunity to disturbances and noise characteristics.

## 2. Time-Correlated Single-Photon Counting

### 2.1. Materials and Methods

**Figure 1:**
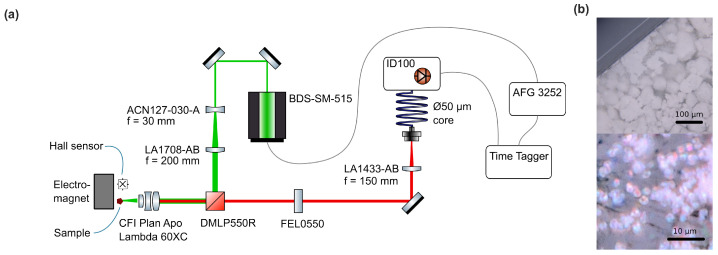
(**a**) Schematic of the optical setup used for lifetime measurements. (**b**) Photograph of the ensemble of microdiamonds in a glass cuvette. The lower panel shows a close-up that reveals individual crystals. A red hue appears due to contrast enhancement.

In Figure 1a, we show a schematic of the TCSPC setup. A ps-Laser (BDS-SM-515, Becker & Hickl GmbH, Berlin, Germany) is used to excite a sample via a dichroic mirror (DMLP550R, Thorlabs, Newton, NJ, USA) and a microscope objective lens (CFI Plan Apo Lambda 60XC, Nikon, Tokyo, Japan). The beam is widened to ~5.3mm (ACN127-030-A and LA1708-AB, Thorlabs, Newton, NJ, USA) and focused by the objective to a beam spot size of 0.42μm. Fluorescence is collected through the same objective and passed through the dichroic mirror and a 550nm long-pass filter (FEL0550, Thorlabs, Newton, NJ, USA). It is focused (LA1433-AB, Thorlabs, Newton, NJ, USA) onto the end facet of a multimode fiber, connected to an avalanche photodiode (APD) detector module (ID100, ID Quantique SA, Acacias—Genève, Switzerland). We use no spatial filtering, so a larger volume than the beam spot size is effectively excited and contributes to the collected fluorescence. The laser is triggered by a function generator (AFG3252, Tektronix Inc., Beaverton, OR, USA), and histograms are acquired by a time-tagging module (Time Tagger 20, Swabian Instruments GmbH, Stuttgart, Germany). Samples are mounted in front of an electromagnet connected to a laboratory power supply. A Hall effect-based sensor (TL493D, Infineon Technologies AG, Munich, Germany) next to the sample is used to measure the applied magnetic field. We measure the instrument response function (IRF) by placing a cover slip in the sample location and acquiring the histogram without the long-pass filter in the beam path. The sample, shown in Figure 1b, is a powder composed of μm-sized diamonds with a high concentration of NV centers, which for the measurements was contained in a glass tube. For this material, we expect an isotropic response to magnetic fields, which has already been demonstrated for the use of fluorescence intensity [6].

Recorded fluorescence histograms are background-corrected by the average count in the first 20ns and fitted using a single- (k = 1) or double-exponential (k = 2) function

(1) $$I_{\mathrm{fl}}\left(t\right)=\sum_{i=1}^{k}a_{k,i}\;e^{-t/\tau_{k,i}}$$

using non-linear least squares (NLLS) analysis. Normalized factors are denoted by ${\hat{a}}_{k,i}=a_{k,i}/\sum_{j=1}^{k}a_{k,j}$. A goodness-of-fit $\chi_{R}^{2}$ used in this work is given by $\chi_{R}^{2}=\chi^{2}/\left(n-p\right)$ with the number of data points *n* and number of free parameters *p*. $\chi^{2}$ is the sum of squared deviations between measured data and fit values, each divided by squared deviations expected for the number of detected photons [18].

### 2.2. Results and Discussion

**Figure 2:**
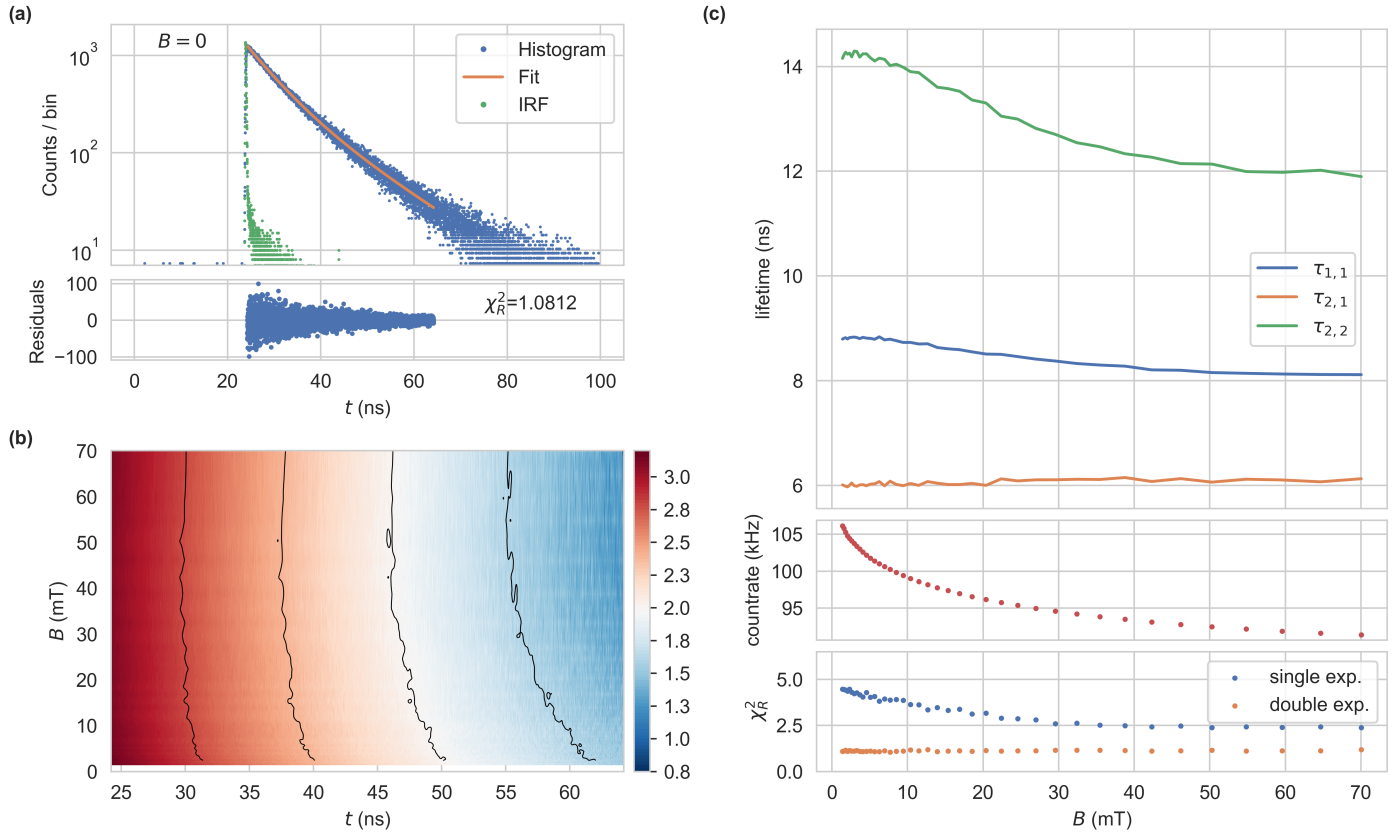
(**a**) Single histogram of a sample composed of NV-rich diamond powder, captured at B=0 (Bin width 10ps, 15s capture time, 8MHz repetition rate, 240μW average power, APD count-rate 81kHz). The average count in the first 20ns of 5.7 counts has been subtracted. The lower pane shows the residuals of a double-exponential fit ($a_{2,1}=848,\tau_{2,1}=6.13\;\mathrm{ns},a_{2,2}=408,\tau_{2,2}=14.54\;\mathrm{ns}$, $\chi_{R}^{2}=1.0812$). For the fit, we use a time span of 40ns, following the maximum. The IRF was captured at the same count-rate at the APD (FWHM = 0.2ns). (**b**) Histograms of the sample at varying magnetic flux densities *B*, shown in the time range used for the fit. A logarithmic color scale is chosen. (**c**) Single and double-exponential fits for the data in (**b**) with fixed ${\hat{a}}_{2,1}=1-{\hat{a}}_{2,2}=0.65$. The three panels show the extracted lifetimes, the count-rate at the APD, and $\chi_{R}^{2}$ for the respective fits, as functions of *B*.

In Figure 2a, we show a single histogram at zero magnetic field, which can be fit well using a double-exponential function resulting in $\chi_{R}^{2}=1.081$ with ${\hat{a}}_{2,1}=0.675,\tau_{2,1}=6.13\;\mathrm{ns},$ ${\hat{a}}_{2,2}=0.325,\tau_{2,2}=14.54\;\mathrm{ns}$. A single exponential function would give a worse fit, with the residuals showing a systematic error instead of a normal distribution around zero. We recorded the magnetic field-dependent fluorescence histograms, shown in Figure 2b and analyzed them using either single- or bi-exponential fits, as shown in Figure 2c. We still see an overall better agreement of the data to a bi-exponential fit and B-field-dependent changes in the decay times, which correlate with the fluorescence intensity. The resolution of two decay time components is challenging if the decay times are of the same order of magnitude [18]. While the data support the acceptance of two decay components, the values of $a_{k,i}$ and $\tau_{k,i}$ are not well determined. To increase the quality of the fit for the application of magnetic field sensing, the fit parameter ${\hat{a}}_{2,1}$ is fixed to $1-{\hat{a}}_{2,2}=0.65$. Similar fits could also be obtained by a positive shift of ${\hat{a}}_{2,1}$ towards higher magnetic fields and a constant short decay time $\tau_{2,1}$, as well as intermediate values. To characterize the influence of B on a measurement quantity *x*, we define the magnetic contrast of the quantity as 1−x(B)/x(B=0) with the saturation value x(B) towards high magnetic fields and its value at B=0. The reduction in fluorescence count-rate shows a magnetic contrast of 13.9% while the larger decay time displays a contrast of 15.2%.

The use of single exponential fits can be found in the literature, where fluorescence lifetimes of 10.0–12.9 ns for ensembles of NV centers [17,19,20,21] and a range of ~10–30ns for single centers [22,23,24,25] are reported. However, even in single NV centers, the existence of a bi-exponential decay has been shown and is attributed to spin sub-levels of the excited-state ^3^E with different lifetimes due to differing non-radiative decay rates to the intermediate singlet states [26,27]. Lifetimes of the sub-levels of 6.3–9.0ns for $m_{S}=\pm 1$ and 12–17.8ns for $m_{S}=0$ have been found [26,27,28,29,30]. When an off-NV-axis magnetic field is applied, a mixing of spin states arises [31]. This magnetic coupling leads to a lifetime of mixed states showing an intermediate value, which has been observed for ensembles of NV centers in bulk diamond [17]. In our measurements, the reduction of the larger decay time by magnetic fields may be explained similarly. In nanodiamonds, a short decay time component is usually attributed to surface or bulk impurities and the larger decay time component to the fluorescence of NV centers [32,33,34,35]. Control or even tuning of these parameters can be achieved by radiation treatment, annealing, and surface termination [33]. Additionally, the crystal size has an influence on the observed lifetimes [35,36], and we expect fluorescence contributions from the neutral charge state ${\mathrm{NV}}^{0}$, which differs in its lifetime from ${\mathrm{NV}}^{-}$ [25,33,37]. Although further investigation is needed, the sample used in this work shows a magnetic field-dependent behavior that can be utilized in a sensing application.

## 3. Frequency Domain Lifetime Measurements

While TCSPC measurements deliver complete histograms, their implementation is technically demanding. Additionally, the acquisition is slow when considering the application of magnetic field sensing. A setup based on the frequency domain measurement is proposed to make use of the change in the excited-state fluorescence lifetime of NV centers for magnetometry. The fluorescence response can be understood as a convolution of the excitation signal with the decay dynamics, acting as a low-pass filter. Instead of monitoring the time-domain decay from a short excitation pulse, we employ harmonic oscillation with a variable frequency. With increasing frequency, the low-pass characteristic will lead to a reduction of the fluorescence amplitude, commonly referred to as demodulation. This is accompanied by a phase shift of the fluorescence signal from 0 to $90^{\circ }$ [18]. In our setup, we sweep the frequency up to 100 MHz and record the system response in both amplitude and phase. This frequency response not only contains the effects of the NV diamond powder but also all electronic and optical components involved in the excitation and recording of the fluorescence. Examples are a frequency-dependent excitation power or the transition time of the signal through the system, leading to a frequency-dependent phase shift. Therefore, we record a reference frequency response at B=0, which is used to calibrate subsequent measurements. The resulting measurements then directly reflect the change in the fluorescence decay dynamics at the application of a magnetic field.

### 3.1. Materials and Methods

**Figure 3:**
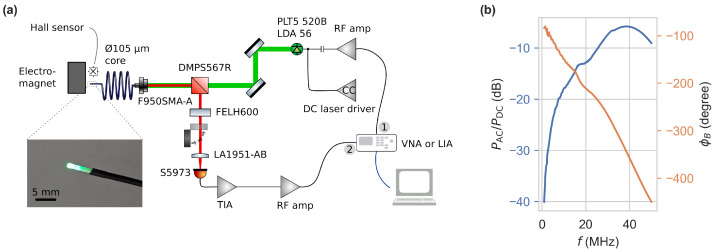
(**a**) Schematic of the optical and electrical setup used for frequency domain measurements. (**b**) Excitation light modulation $P_{\mathrm{AC}}/P_{\mathrm{DC}}$ measured via reflections with the long-pass filter removed and phase difference $\phi_{B}$ between excitation and reception signal.

The optical and electrical setup for frequency domain measurements is depicted in Figure 3a. A 520nm laser diode (PLT5 520B, ams-OSRAM AG, Premstaetten, Austria) is driven by a constant current source based on a laser driver integrated circuit (NZN, iC-Haus GmbH, Bodenheim, Germany). Additionally, it is modulated by an AC-coupled radio frequency (RF) amplifier (35dB, 1–700MHz, 3.2W) at an input power of −9dBm. The excitation light is collimated, propagates through a dichroic beam splitter (DMPS567R, Thorlabs, Newton, NJ, USA), and is coupled to a 105μm core diameter fibre. The end facet of the fiber is coated with NV-rich diamond powder in glue, with crystal sizes much smaller than the facet diameter. The material is comparable in NV density to the material used in [6]. The fiber sensor was manufactured by Quantum Technologies GmbH in collaboration with the Leibniz Institute of Surface Engineering. The fluorescence is collected through the same fiber, passing through a long-pass filter and focused (FELH600 & LA1951-AB, Thorlabs, Newton, NJ, USA) onto a Si-photodiode (S5973, Hamamatsu Photonics K.K., Hamamatsu City, Japan). The photodiode is part of a trans-impedance amplifier (TIA) based on an OPA847 operational amplifier (300MHz, 1.2kΩ). Its output is passed through a low-noise RF amplifier (20dB, 0.1–2000MHz) to a PicoVNA 106 vector network analyzer (VNA) (Pico Technology, St Neots, United Kingdom) or HF2LI lock-in amplifier (LIA) (Zurich Instruments AG, Zurich, Switzerland), which also drives the laser diode modulation. An electromagnet, connected to a PC-controlled power supply, is being monitored by a Hall effect sensor and enables the application of magnetic flux densities from 0 to ~120mT.

The modulation of the excitation light can be described by an offset $P_{\mathrm{DC}}$ and amplitude of the AC component $P_{\mathrm{AC}}$. To assess the modulation, we measured these via reflection of the pump beam after removing the long-pass filter at the DC-coupled TIA output. Their ratio $P_{\mathrm{AC}}/P_{\mathrm{DC}}$ is depicted in Figure 3b. It shows a high pass characteristic and overall non-constant ratio originating from the RF amplifier for laser modulation. The laser diode output power measured before the dichroic mirror varies between 33 and 37mW, depending on the modulation frequency. The use of a VNA, in particular, requires the calibration of the scattering parameter $S_{21}$, i.e., the magnitude and phase relationship of the signals of ports one and two. Therefore, *isolation* and *through* was calibrated by either blocking the fluorescence path or passing it unrestricted to the photodetector, ensuring no magnetic field was applied to the sensor head. This way, the instrument automatically corrects for the reference frequency response.

### 3.2. Results and Discussion

**Figure 4:**
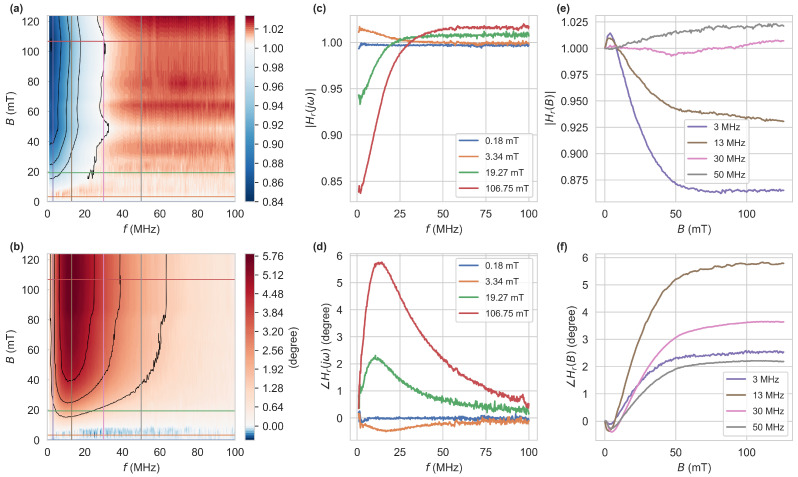
Measurements of the complex transfer function $H_{r}=\left|H_{r}\right|\exp\left(j∠H_{r}\right)$. The top and bottom rows show the magnitude $|H_{r}|$ and phase $∠H_{r}$, respectively. $H_{r}$ is the transfer function H(jω,B), relative to H(jω,B=0). (**a**–**d**) were obtained directly by sweeping the modulation frequency using a VNA, which was calibrated at B=0. For (**e**,**f**), a LIA at the respective modulation frequencies was used to capture magnitude and phase. The data were subsequently normalized to the data point at B=0.

Figure 4a,b show the magnitude and phase of the fluorescence signal as a function of modulation frequency and applied magnetic field (acquired with the VNA), relative to B=0. The horizontal and vertical lines correspond to the other measurements shown in the figure. On the left edge at near zero frequency, we see a relative magnetic contrast of 16% in magnitude, which has already been observed and used for all-optical magnetometry [6,38]. At higher modulation frequencies, this behavior changes significantly. The magnitude for all B>0 declines, and a response of the phase to magnetic fields emerges. The phase displays a maximum in magnetic contrast of $5.8^{\circ }$ at 13MHz. Towards higher modulation frequencies, both quantities decrease in magnetic contrast, and the magnitude rises above one at an inflection point around 30MHz. At this frequency, the magnitude is nearly independent of the magnetic field (cf. pink trace in Figure 4e) while the phase still shows a magnetic contrast of $3.6^{\circ }$. This could potentially be used to calibrate the optical path. At lower optical excitation powers, we observe a similar behavior with the difference of a lower signal-to-noise ratio and a decrease in magnetic contrast of both measurement quantities. Many fiber sensors were produced and show good reproducibility in fluorescence intensity and response to static magnetic fields, but lifetime measurements were only performed for one fiber. We also expect lifetime measurement results to be similar for different fiber sensors.

We operate the system at optical excitation powers for which we expect no saturation behavior [39,40]. In this linear regime, the fluorescence can be understood as a convolution of the excitation signal with the decay dynamics, acting as a low-pass filter. Considering single- and bi-exponential fluorescence decays, their respective transfer functions H(s) can be written as

(2) $$H\left(s\right)=\frac{a_{2,1}}{s+\frac{1}{\tau_{2,1}}}+\frac{a_{2,2}}{s+\frac{1}{\tau_{2,2}}}$$

by the Laplace-transform of Equation (1) with s=σ+jω, where for a single exponential approach $a_{2,2}=0$. To model the measurements in Figure 4 the normalization to ${H\left(s\right)|}_{B=0}$ at B=0 is considered, resulting in $H_{r}\left(s\right)=H\left(s\right){{|}_{B}/H\left(s\right)|}_{B=0}$. Magnitude $|H_{r}\left(s=j\omega \right)|$ and phase $∠H_{r}\left(s=j\omega \right)$ can then be obtained from this. To reproduce the inflection point around 30MHz and a magnitude greater one at high modulation frequencies, at least a bi-exponential approach is needed. This becomes apparent when considering

(3) $$H_{r}\left(s\right)=\left(1+\frac{\Delta a}{a_{1,1}}\right)\frac{s\left(\tau_{1,1}+\Delta \tau \right)+1+\frac{\Delta \tau }{\tau_{1,1}}}{s(\tau_{1,1}+\Delta \tau )+1}$$

for a single exponential approach, with $\tau_{1,1}{{|}_{B}=\tau_{1,1}|}_{B=0}+\Delta \tau$, $\;a_{1,1}{{|}_{B}=a_{1,1}|}_{B=0}+\Delta a$ and Δτ, Δa being functions of *B*. $|H_{r}\left(s=j\omega \right)|$ can then only be strictly greater or less than one for Δτ>0 or Δτ<0, respectively. Therefore, a single exponential approach is insufficient to model this behavior. The data can, however, be fit well by $H_{r}\left(s\right)$ with constant factors ${\hat{a}}_{2,1}=1-{\hat{a}}_{2,2}=0.65$ resulting in $\Delta \tau_{2,1}=-2.52\;\mathrm{ns}$ and $\Delta \tau_{2,2}=0.19\;\mathrm{ns}$ at B>100mT. These findings agree well with the fits to the time-domain histograms from Figure 2. Analogous to the time domain fits, similar fitting transfer functions could be obtained from a constant $\tau_{2,2}$ and shift in ${\hat{a}}_{k,i}$, or intermediate values. The exact dynamics do not need to be deduced from these measurements, as the behavior, especially the reduction in the larger decay time, allows the utilization in a sensing application. This may be done by capturing the complete frequency response to calculate the applied magnetic field. Alternatively, we record the magnitude and phase at one excitation frequency with a LIA, yielding a higher acquisition speed.

**Figure 5:**
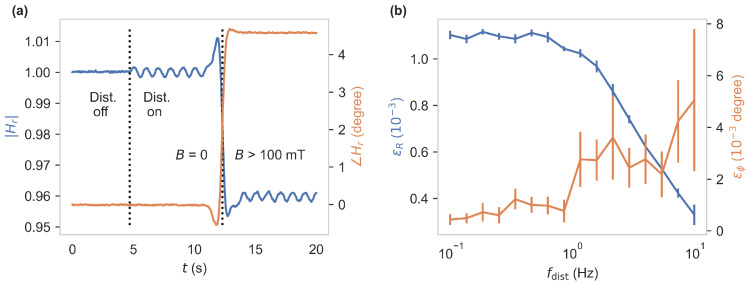
(**a**) Measurement quantities $|H_{r}|$ and $∠H_{r}$ at f=13MHz (4th order, 3.89Hz low-pass demodulator). The excitation *disturbance* modulation with frequency $f_{\mathrm{dist}}=1\;\mathrm{Hz}$ was switched on at t=5s. For comparison to the magnetic contrasts of both quantities, a magnetic field of >100mT has been applied at t=12s. (**b**) Root-mean-square values of the AC components of magnitude and phase at the disturbance frequency $f_{\mathrm{dist}}$ in a range of 0.1–10Hz. Here, a bias magnetic field corresponding to half of the magnetic contrast was applied.

The key advantage of using the phase instead of the magnitude lies in its immunity to disturbances that affect the intensity of the signal. These include laser intensity noise and thermal drifts in the optical alignment, as well as motion in the fiber, causing fluctuations in the excitation and the returning fluorescence. We use an LIA to monitor and compare magnitude and phase. Hence, we set the excitation frequency to f=13MHz, where the phase shows the maximum contrast. Now, we test the immunity. We add a small modulation of the laser intensity in a frequency range up to 10Hz. This translates to an excitation intensity modulation, which can be expected in an application through attenuation by, e.g., bending of the fiber. This artificial disturbance is achieved by a Liquid Crystal Light Valve (LCLV) placed between the adjustment mirrors and the dichroic beam splitter, which can be continuously modulated in its transmission. Figure 5a shows magnitude $|H_{r}|$ and phase $∠H_{r}$ in a time range of 20s. The additional *disturbance* modulation with frequency $f_{\mathrm{dist}}=1\;\mathrm{Hz}$ is switched on at t=5s which leads to an excitation intensity of 8.88mW fluctuating with ±0.17%. Additionally, at t=12s, a magnetic field of >100mT was applied for easy comparison of both measurement quantities to their magnetic contrast. A lower impact on $∠H_{r}$ relative to its contrast is obvious.

To measure these errors in a wider frequency range and at a higher precision, the LIAs 13MHz demodulator outputs *R* or ϕ were fed back into a second demodulator, running at $f_{\mathrm{dist}}$ and therefore measuring the root-mean-square (RMS) value of the disturbance modulation components. We set the first demodulator low-pass filter to 1kHz, making sure the additional disturbance component passes to the second one without attenuation. Additionally, a bias magnetic field corresponding to half of the magnetic contrast was applied to bring the system to a point of operation that is realistic during an application. We then swept $f_{\mathrm{dist}}$ in a frequency range of 0.1Hz to 10Hz and recorded the second demodulator output. This second output is a measure for the low-frequency disturbances in magnitude or phase, which we denote by $\epsilon_{R}$ or $\epsilon_{\phi }$, respectively. They are shown in Figure 5b. For lower frequencies $\epsilon_{R}\approx 1.1\cdot 10^{-3}$, which is 2.3% of the magnetic contrast of $|H_{r}|$. In comparison $\epsilon_{\phi }\approx 0.35\cdot 10^{-3}\mathrm{degree}$, a disturbance of only 0.02%, relative to the magnetic contrast of $∠H_{r}$. Above $f_{\mathrm{dist}}=1\;\mathrm{Hz}$, the LCLV drops off in its modulation amplitude, leading to a reduction in $\epsilon_{R}$ and a higher error in the measurement of $\epsilon_{\phi }$. This is caused by the slow response of the LCLV, and we expect the immunity to persist at higher frequency disturbances.

**Figure 6:**
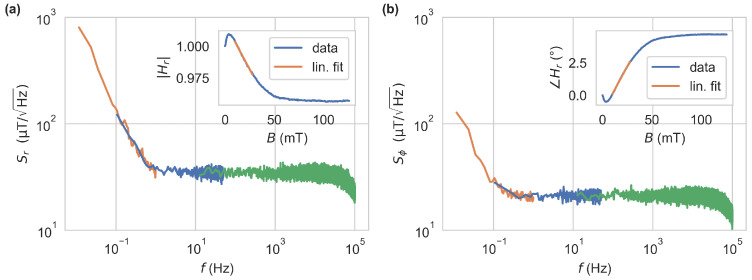
Noise spectral densities based on magnitude $S_{r}$ (**a**) and phase $S_{\phi }$ (**b**). The excitation frequency was set to f=13MHz, and a bias field of B=20mT was applied. Data were recorded by the LIA with the low-pass filter set to fourth order at corner frequencies of 88kHz, 200Hz and 2Hz, corresponding to the green, blue and orange traces. They have been subsequently converted to magnetic field values using the linear fits drawn in the respective insets and converted to spectral densities by Welch’s method.

The sensitivity achievable by both approaches, i.e., magnitude or phase as a measurement quantity, was further investigated. Therefore, a bias magnetic field of B=20mT was applied, and data were recorded with the LIA set to f=13MHz and subsequently converted to magnetic field values using linear fits to $|H_{r}\left(B\right)|$ and $∠H_{r}\left(B\right)$, depicted in the insets of Figure 6. The respective noise spectral densities are shown in Figure 6 reveal a white noise region above 1Hz with sensitivities of $s_{r}=35\;\mu T/\sqrt{\mathrm{Hz}}$ and $s_{\phi }=20\;\mu T/\sqrt{\mathrm{Hz}}$. The excitation light source was operated at 11.5mW. In this case, the fluorescence signal leads to a photodiode current with DC component $I_{\mathrm{DC}}=0.213\;\mu A$ and amplitude of the AC component of $I_{\mathrm{AC}}=0.171\;\mu A$ at f=13MHz. This results in shot-noise-limited sensitivities (SNLS) of $s_{r,\mathrm{snl}}=1.13\;\mu T/\sqrt{\mathrm{Hz}}$ and $s_{\phi ,\mathrm{snl}}=0.95\;\mu T/\sqrt{\mathrm{Hz}}$. The SNLS in LIA-based setups are derived in Appendix A. The difference in the achieved sensitivities is largely based on the TIA’s output noise spectral density, being limited by the feedback resistor’s thermal noise of $4.4\;\mathrm{nV}/\sqrt{\mathrm{Hz}}$ and the operational amplifier’s input current noise contributing $3.24\;\mathrm{nV}/\sqrt{\mathrm{Hz}}$. These dominate the shot noise, contributing only $0.336\;\mathrm{nV}/\sqrt{\mathrm{Hz}}$ at the given photocurrent. The same measurement at f=3MHz leads to sensitivities of $s_{r}=12\;\mu T/\sqrt{\mathrm{Hz}}$ and $s_{\phi }=43\;\mu T/\sqrt{\mathrm{Hz}}$, which are caused by different magnetic contrasts at this frequency. Additional 1/f components dominate the spectrum below 1Hz in both approaches. These are attributed largely to the excitation and detection electronics.

## 4. Conclusions and Outlook

The fluorescence lifetime of a high-NV-density microdiamond powder has been investigated, showing a bi-exponential behavior with a magnetic contrast of the larger decay time of 15.2%. This constitutes a non-intensity-based quantity for magnetic field sensing, which is used in a MW-free fiber-based setup. The bi-exponential function is interpreted as a sum of two first-order low-pass filters, which change their gain and corner frequencies upon application of a magnetic field. This is accompanied by a change in phase, which shows a maximum in magnetic contrast of $5.8^{\circ }$ at 13MHz. We employ an LIA at this modulation frequency and use the phrase as a non-intensity quantity for magnetic field sensing. We realize a 100-times-higher immunity to intensity fluctuations because we avoid the misinterpretation of changes in fluorescence intensity as changing magnetic fields. In the current state, the system shows comparable realized sensitivities and expected SNLS for the magnitude and phase approaches. The construction of the TIA leaves room for improvement upon narrowing the required bandwidth to a small band around 13MHz to realize a sensitivity closer to the SNLS, which we estimate to $s_{\phi ,\mathrm{snl}}=950\;\mathrm{nT}/\sqrt{\mathrm{Hz}}$ at an excitation power of 11.5mW. In shot-noise-limited measurements, the signal-to-noise ratio scales with the square root of the detected photons per time. However, we are limited by significant increases in the excitation power and, therefore, fluorescence intensity by heat introduced into the fiber head. Next to the shot noise, the achievable sensitivity in our setup is primarily determined by the derivatives of the measurement quantities $\delta |H_{r}|/\delta B$ and $\delta ∠H_{r}/\delta B$, which increase with excitation power. We expect a saturation towards higher powers, which has been observed for the magnitude before [6].

Current state-of-the-art NV-based sensors realize impressive sensitivities, reaching $0.9\;\mathrm{pT}/\sqrt{\mathrm{Hz}}$ for NV ensembles [41] and $170\;\mathrm{pT}/\sqrt{\mathrm{Hz}}$ for single NV centers [42]. They are based on optically detected magnetic resonance, requiring MW excitation. Other all-optical setups also achieve higher sensitivities at 0.45–$6\;\mathrm{nT}/\sqrt{\mathrm{Hz}}$, using the GSLAC [10,11,12] or ~$10\;\mathrm{nT}/\sqrt{\mathrm{Hz}}$, utilizing the zero-field features [14]. Both are based on narrowband features, requiring stable bias magnetic fields with accurate alignment. All of these setups need bulky and costly infrastructure bound to a laboratory environment. Our setup, in contrast, can potentially be realized in a more compact and less costly manner. It also shows a consistent sensitivity in a higher magnetic field range of approximately 10–40mT, making it more universally applicable. These findings establish the basis for the application of fluorescence lifetime in all-optical, low-noise, and robust magnetometry. The integration of this approach holds promise for advancing magnetic field sensing capabilities, particularly in applications where conventional methods are limited by a galvanic connection, metallic components in the sensing volume, or the interaction with MW radiation.

## Data Availability

Data underlying the results presented in this paper are not publicly available at this time but may be obtained from the authors upon reasonable request.

## Abbreviations

The following abbreviations are used in this manuscript:

NV	Nitrogen vacancy
MW	Microwave
TCSPC	Time-correlated single-photon counting
APD	Avalanche photodiode
IRF	Instrument response function
FWHM	Full width at half maximum
NLLS	Non-linear least squares
AC	Alternating current
RF	Radio frequency
TIA	Trans-impedance amplifier
VNA	Vector network analyzer
LIA	Lock-In amplifier
DC	Direct current
LCLV	Liquid Crystal Light Valve
RMS	Root-mean-square
SNLS	Shot-noise-limited sensitivity

## Appendix A

Shot noise in optical devices arises due to fluctuations in the number of photons detected per time because they occur independently of each other, and sets a lower bound to the achievable sensitivities in the presented setup. To determine its impact on intensity- or phase-based all-optical systems, first, the influence of noise on amplitude and phase in general has to be determined. The following considerations are based on [43,44,45]. A lock-in amplifier output signal can be considered to be

(A1) $$l\left(t\right)=x\left(t\right)+jy\left(t\right)=r\left(t\right)e^{j\phi (t)}$$

with in-phase x(t) and quadrature y(t) components or amplitude r(t) and phase ϕ(t). For each component, the mean and standard deviation are given by μ and σ, subscripted by the component name. The output is considered to be the response of a homodyne detector to a sinusoidal signal with additional noise. It can be assumed that the noise components of in-phase and quadrature are

1. Gaussian random processes with zero-mean, normal distributions,
2. statistically independent, and
3. contain identical energy, i.e., $\sigma_{x}=\sigma_{y}=\sigma$.

with the parameter $\mu =\sqrt{\mu_{x}^{2}+\mu_{y}^{2}}$, the amplitude is then described by the Rician distribution

(A2) $$f\left(r\right)=\frac{r}{\sigma^{2}}e^{-\frac{r^{2}+\mu^{2}}{2\sigma^{2}}}I_{0}\left(\frac{r\mu }{\sigma^{2}}\right),\;\;r\geq 0$$

with the modified Bessel function of the first kind with order zero $I_{0}$. The phase distribution is given by

(A3) $$f\left(\phi \right)=\frac{e^{-\frac{\mu^{2}}{2\sigma^{2}}}}{2\pi }\left(1+\frac{\mu }{\sigma }\sqrt{\frac{\pi }{2}}\mathrm{erfcx}\left(-\frac{\mu }{\sigma }\sqrt{2}\cos\left(\phi -\theta \right)\right)\right),\;\;-\pi \leq \phi \leq \pi$$

with $\theta =\tan^{-1}\left(\mu_{y}/\mu_{x}\right)$ and the scaled complementary error function erfcx. While for the amplitude distribution, the mean $\mu_{r}$ and standard deviation $\sigma_{r}$ are well defined, there are no closed-form formulas for the phase distribution. So, in this work, they are obtained computationally by $\mu_{\phi }=\int_{-\infty }^{\infty }\phi f\left(\phi \right)d\phi$ and $\sigma_{\phi }^{2}=\int_{-\infty }^{\infty }\phi^{2}f\left(\phi \right)d\phi -\mu_{\phi }^{2}$. The nature of the erfcx function necessitates an arbitrary-precision floating-point arithmetic library, like mpmath [46].

In the given application, the photodiodes output current is composed of a DC offset $I_{\mathrm{DC}}$ and AC component of amplitude $I_{\mathrm{AC}}$, resulting in a root-mean-square current $I_{\mathrm{rms}}=\sqrt{I_{\mathrm{DC}}^{2}+\frac{I_{\mathrm{AC}}^{2}}{2}}$ contributing a shot noise density of $I_{\mathrm{sn}}=\sqrt{2qI_{\mathrm{rms}}}$ with the elementary charge *q*. With $\mu =I_{\mathrm{AC}}/\sqrt{2}$ and $\sigma =I_{\mathrm{sn}}$ the shot-noise-limited sensitivity for the magnitude can then be defined as

(A4) $$s_{r,\mathrm{snl}}={\left|\frac{\delta r(B)}{\delta B}I_{\mathrm{AC}}\right|}^{-1}\sigma_{r}$$

in units of $T/\sqrt{\mathrm{Hz}}$. Here $r\left(B\right)=|H_{r}\left(s=j\omega ,B\right)|$ is the change in magnitude relative to the case of B=0 at a fixed excitation frequency ω=2πf. Similarly for the phase

(A5) $$s_{\phi ,\mathrm{snl}}={\left|\frac{\delta \phi (B)}{\delta B}\right|}^{-1}\sigma_{\phi }$$

with $\phi \left(B\right)=∠H_{r}\left(s=j\omega ,B\right)$. Please note that in the last steps, $\sigma_{r}$ and $\sigma_{\phi }$ are spectral densities instead of RMS values. The argument stays valid because the shot noise is white, i.e., constant over frequency.
